# Isolation and Physiomorphological Characterization of* Escherichia coli* O157:H7-Infecting Bacteriophages Recovered from Beef Cattle Operations

**DOI:** 10.1155/2017/7013236

**Published:** 2017-10-16

**Authors:** Pushpinder Kaur Litt, Divya Jaroni

**Affiliations:** Department of Animal Science and Food and Agricultural Products Center, Oklahoma State University, 148 FAPC, Monroe St., Stillwater, OK 74078, USA

## Abstract

Bacteriophages, recovered from beef cattle environment and specifically targeting* Escherichia coli* O157:H7, were examined for their physiological and morphological characteristics. Degree of bacterial lysis and host range of isolated bacteriophages was determined against 55 isolates of* E. coli* O157:H7. Morphology of phages was examined under transmission electron microscope. Phage growth parameters, particularly rate of adsorption, rise period, latent period, and burst size were also determined. The stability of isolated phages was tested at acidic and alkaline pH, at high temperatures, and in cold storage. A total of 7 phages were isolated which showed lytic activity against 50 out of 55 isolates of* E. coli* O157:H7. Based on the morphology, phages were classified into Myoviridae or Siphoviridae family. Phages had a rise period between 19 and 40 min, a short latent period between 12 and 30 min, and a large burst size (89–631 virions per infected cell), indicating high lytic activity. Phages remained stable for 24 h at a wide pH (1–11) and temperature range (40–60°C) and for 90 d in cold storage. Characterization of bacteriophages, with a diverse host range of* E. coli* O157:H7, could aid in the development of effective biocontrol strategies for this pathogen in the food industry.

## 1. Introduction

*Escherichia coli* O157:H7 is an important foodborne pathogen that lives commensally in the rumen of cattle and other food animals such as sheep and goat [1–5]. Additionally, deer, horses, dogs, and birds can also transiently harbor this pathogen [4, 6–8]. Direct or indirect contact with animals or manure of animals carrying* E. coli* O157:H7 could mediate its transfer to water and food products, which could result in human infections upon consumption. In a typical year in the United States (US),* E. coli* O157:H7 causes an estimated 63,000 foodborne illnesses, 2,100 hospitalizations, and 20 deaths, imposing an economic burden of $271 million [9, 10]. Advanced stages of pathogen infection include hemorrhagic colitis, which could progress into hemolytic uremic syndrome. Severe complications are characterized by renal failure, hemolytic anemia, and thrombocytopenia, which could be fatal [11–13]. Some high-risk food commodities associated with these illnesses include beef and meat products, fresh produce, unpasteurized apple juice, and dairy products [13–28].

Reduction of* E. coli* O157:H7 at the preharvest and processing level could play a significant role in preventing the introduction of this pathogen into the food chain. Various approaches, such as diet and probiotic therapy, vaccination, and antibiotics, against* E. coli* O157:H7 have been evaluated to control its prevalence in food animals [3, 10, 29]. However, recolonization of previously infected animals, with the same or different strains of* E. coli* O157:H7, limits the effectiveness of these strategies [3, 30]. Similarly, at the processing level, various control measures such as organic acids, chlorine, or other antimicrobial washes have been used to control* E. coli* O157:H7 persistence in foods [31–34]. However, these interventions remain insufficient, as is evident from the continuing foodborne illness outbreaks and food recalls associated with this pathogen. It is therefore important to develop effective alternatives to control* E. coli* O157:H7 in the food and animal industry.

Targeted use of bacteriophages against* E. coli* O157:H7 could reduce this problem. Bacteriophages are ubiquitous, naturally abundant viruses, which invade and kill specific bacteria. They are recognized as gastrointestinal (GI) commensal microorganisms and have been isolated from various sources [35, 36]. Almost a century ago, d'Herelle [37] demonstrated the efficacy of bacteriophages against human pathogens. However, more recently, due to the emergence of antibiotic-resistant microorganisms, the long-lost phage therapy has regained interest for use against foodborne pathogens. Under* in vitro *conditions, virulent bacteriophages have shown the potential for selective elimination of* E. coli* O157:H7 [38, 39]. Additionally, studies in sheep have shown that gut colonization by* E. coli* O157:H7 can be prevented by oral administration of O157:H7-infecting phages [39]. Efficacy of bacteriophages against* E. coli* O157:H7 has also been documented in various foods such as cantaloupe, spinach, lettuce, tomato, broccoli, and ground beef [40–42]. In a study by O'Flynn et al. [43], a cocktail of* E. coli* O157:H7-specific bacteriophages completely reduced pathogen counts on the surface of beef steaks. Additionally, studies have shown that phages can effectively eliminate* E. coli* O157:H7 in biofilms on various food-processing surfaces such as stainless steel and high-density polyethylene [42, 44]. Even though studies have demonstrated the potential of phage-based technologies to control* E. coli* O157:H7, very little is known about the morphology, physiology, and characteristics of specific phages. At the same time, selection of effective bacteriophages for food and animal industry applications also requires an understanding of their survival under various stress conditions. The present study was aimed at isolating* E. coli* O157:H7-specific bacteriophages from beef cattle operations and determining their physiomorphological characteristics, including survival under various pH, high temperatures, and cold storage conditions.

## 2. Materials and Methods

### 2.1. Bacterial Cultures

Isolation of bacteriophages was carried out using the host strain,* E. coli* O157:H7 ATCC 43895 (Table 1). Host range of isolated phages was determined using 55* E. coli* O157:H7 isolates (Table 1). Two of these isolates were clinical strains (ATCC 43895, ATCC 43888) while the rest were wild-type (WT) isolates, retrieved from our laboratory culture collection, originally isolated from bovine feces or beef cattle farm environment [45]. Prior to an experiment, overnight culture of each* E. coli* O157:H7 isolate was prepared in tryptic soy broth (TSB; Bacto™, BD, Sparks, MD) and incubated statically at 37°C for 18 h.

### 2.2. Bacteriophage Isolation

Over a period of two years, water (*n* = 67) and bovine fecal (*n* = 60) samples were collected from beef cattle operations in Oklahoma, during the summer months, and used for isolation of bacteriophages specific to* E. coli* O157:H7 [45]. Water (10 mL) and fecal (10 g) samples were enriched in 25 mL double strength NZ-amine casamino acid yeast extract sodium chloride magnesium sulfate (NZCYM; RPI Corp, IL) broth for 18 h at 37°C, along with 10 mL of host bacterial culture (*E. coli* O157:H7 ATCC 43895). After incubation, 2 mL suspension was centrifuged at 12,000 rpm for 10 min to remove cellular debris and fecal material. The supernatant was filtered using 0.45 *µ* syringe filter (EMD Millipore Millex™, Carrigtwohill, Ireland) and the filtrate plated on NZCYM agar (Fisher Scientific, NJ) via double-layer agar method, as described by Sambrook et al. [46]. The presence of phage was confirmed by plaque formation on the agar plate and the phage further concentrated by the method described by Chandra et al. [47]. Briefly, an overnight culture (100 *µ*L) of* E. coli* O157:H7 was suspended in molten (0.75%) NZCYM agar and poured onto an NZCYM agar plate which was allowed to solidify for 2–5 min. The plate was then streaked with the phage, as horizontal and vertical lines, using a sterile platinum loop and incubated at 37°C for 18–20 h. After incubation, 5 mL SM buffer (10 mM Tris–HCl, pH 7.5; 100 mM NaCl; 10 mM MgSO_4_; Fisher Scientific, NJ) was poured onto the agar plates to elute any plaques along the lines. The agar containing the plaques was then scraped with a sterile platinum loop to release the phages and the elution was centrifuged at 12,000 rpm for 15 min. The resulting supernatant was filtered using a 0.22 *µ* syringe filter, to which 0.1% chloroform (Fisher Scientific, NJ) was added to make a phage working-stock solution which was stored at 4°C until further use. Plaque size (mm) of individual phages was determined by performing plaque assay [48] and by measuring their diameter using Vernier Calipers (Fisherbrand™ Traceable™ Digital Calipers, NJ).

Prior to an experiment, phage titers were determined (Table 2), as plaque forming units (PFU) mL^−1^, by serially diluting the phage working-stock in phosphate buffered saline (PBS; pH 7.4; sodium chloride, Fisher Scientific, NJ; potassium chloride, sodium phosphate monobasic and sodium phosphate dibasic, Sigma-Aldrich, MO) and by performing plaque assay [48].

### 2.3. Bacteriophage Host Range

**Figure 1:**
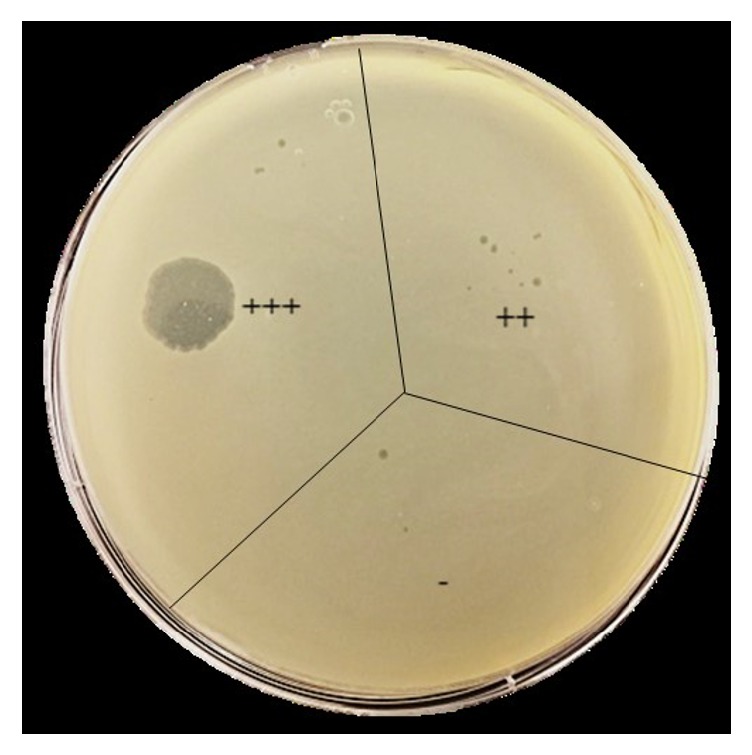
Representative NZCYM agar plate showing the three categories of phage spots: clear (+++), turbid (++), and no reaction (—), on the lawn of host* E. coli* O157:H7.

Host range of all the phages was tested against 55* E. coli* O157:H7 isolates (Table 1), using spot-on-lawn assay [48]. Overnight culture (100 *µ*L) of each bacterial isolate was added to 5 mL of molten (0.75%) NZCYM agar and was overlaid onto an NZCYM agar plate. Ten microliters of the respective phage solution (~10^8^ PFU mL^−1^) was spotted onto each bacterial agar plate. The plates were incubated at 37°C for 18 h and examined for clear zones on the bacterial lawn, to determine the host range of the respective phages. Bacterial sensitivity to a bacteriophage was established by bacterial lysis at the spot where the phage was deposited. Based on the degree of clarity on the bacterial lawn, the spots were differentiated into three categories: clear (+++), turbid (++), or no reaction (—) (Table 1 and Figure 1).

### 2.4. Bacteriophage Morphology

Phage morphology was determined using transmission electron microscopy. Sample was prepared by suspending an overnight culture (100 *µ*L) of* E. coli* O157:H7 in 5 mL molten (0.75%) NZCYM agar and was poured onto a fresh NZCYM agar plate. Phage stock (prepared above) was serially diluted in SM buffer supplemented with 1 M calcium (SM-Ca; Fisher Scientific, NJ). 10 *µ*L of each dilution was spotted onto the bacterial agar plate (prepared above) and incubated at 37°C for 18 h. Following incubation, phages were eluted in 10 *µ*L SM-Ca by carefully pipetting up and down (7–10 times) onto the webbed spot. Each eluted phage sample was negatively stained with 2% phosphotungstic acid (Emsdiasum, Hatfield, PA) on carbon-coated grids (Emsdiasum, Hatfield, PA) and examined under the transmission electron microscope (TEM; JEM-2100TEM, JEOL). Morphology (shape), capsid diameter (nm), and tail length (nm) of the phages were determined by electron micrographs at a magnification of 50,000x (Oklahoma Technology and Research Park Venture, Oklahoma State University, Stillwater, OK).

### 2.5. Bacteriophage Stability at Acidic and Alkaline pH, at High Temperatures, and in Cold Storage

Stability of isolated phages was tested against a wide pH (1–11) and temperature (−80 to 90°C) range to determine their survival under acidic, alkaline, high temperature, and cold storage conditions. All phage stability studies were conducted with a phage population of about 10^8^ PFU mL^−1^ (Table 2).

Stability studies for acidic and alkaline conditions were conducted according to the methods described by Niu et al. [49]. Briefly, phages were suspended in PBS adjusted with 1 M NaOH or HCl (Fisher Scientific, NJ), to yield a pH range of 1–11, and incubated at 37°C to determine their survival at 1, 2, 4, 6, 12, and 24 h.

For thermal stability [50], working stock of each phage (100 *µ*L) was suspended in 900 *µ*L PBS and the suspension was incubated at 40, 60, 70, and 90°C for 60 min in a dry bath (Fisher Scientific, IA). Phage population at each temperature was determined every 10 min during the 60-minute incubation.

For cold storage stability [50], 1 mL stock solution of each isolated phage was stored at 4, −20, and −80°C and sampled at 0, 1, 30, 60, and 90 d. Surviving phage populations for each study were determined by serially diluting the samples in PBS and plating on NZCYM agar, using double-layer agar method.

### 2.6. Phage Adsorption and One-Step Growth Kinetics

Phage adsorption assay [51] was performed to determine the time taken by the phages to adsorb to the host bacterial surface. Their rise period and latent period, along with their burst size, were determined through one-step growth kinetics study. Overnight culture (1 mL) of host* E. coli* O157:H7 (10^9^ CFU mL^−1^) was centrifuged at 12,000 rpm for 2 min. After discarding the supernatant, the pellet was resuspended in 900 *µ*L PBS and 100 *µ*L of the phage-stock solution (10^8^ PFU mL^−1^) added to the suspension to achieve a 0.1 multiplicity of infection (MOI). This bacteria-phage suspension was used for the adsorption and one-step growth kinetics study.

For phage adsorption study, the bacteria-phage suspension was incubated at 37°C for 80 min. After taking an initial sample at 0 min, a 100 *µ*L aliquot was sampled every 20 min by adding to 900 *µ*L PBS and centrifuging for 2 min at 12,000 rpm. The supernatant containing unadsorbed phages was filtered through 0.2 *µ* filter and plated using double-layer agar technique. Percent of unadsorbed phages, at each 20-minute interval, was calculated as the ratio of PFU mL^−1^ in the supernatant to the initial PFU mL^−1^ at 0 min. The percent of adsorbed phages was determined by subtracting the percent of unadsorbed phages from 100.

For one-step growth experiment, the bacteria-phage suspension was incubated at 37°C for 10 min to allow the phages to adsorb to the bacterial cell [52]. After incubation, the suspension was centrifuged at 13,000 rpm for 1 min and the supernatant subjected to plaque assay to determine the titer of the unadsorbed phages. The pellet containing infected bacterial cells was immediately resuspended in 10 mL of prewarmed (37°C) TSB and, after taking an initial sample (100 *μ*L) at 0 min, incubated at 37°C for 60 min. To obtain the one-step growth curve, a 100 *μ*L sample was collected every 5 min, serially diluted, and plated on NZCYM agar using double-layer agar method. The rise period and latent period, as well as the burst size of each phage, were calculated by fitting the one-step growth curve into a 4-parameter sigmoidal model [53] as follows: *P*_*t*_ = *P*_*o*_ + *α*/(1 + *e*^−((*t* − *t*_*r*_)/*b*)^), where *P*_*t*_ was the log phage-density (PFU mL^−1^) at time *t* (min), *P*_*o*_ was the initial log phage-density at time 0 (min), *t*_*r*_ was the rise period (min), *α* was the log density (PFU mL^−1^) of virions per infected cell, and *b* was the maximum rate of exponential phage growth (min^−1^). The burst size (PFU cell^−1^) and latent period (min) were calculated as 10^*α*^ and *t*_*r*_ − 1/*b*, respectively. The latent period was defined as the time-interval between the end of adsorption and the beginning of the first burst (i.e., the constant period), indicated by minimal increase in phage titer [54, 55]. The rise period (after latent period) was identified as the time over which infected bacterial cells lysed, with significant increase in phage titer [54]. The burst size was defined as the maximum yield of phages obtained from the appearance of first phage progeny until complete bacterial cell lysis [48, 56].

### 2.7. Statistical Analysis

All the experiments were repeated three times and the mean values of the three replicates obtained. Surviving phage populations, obtained in each study, were converted to log_10_ PFU mL^−1^. Statistical analysis was performed to determine the effect of pH and high temperature on phage survival. Data were analyzed to determine the analysis of variance (ANOVA) using Duncan's multiple range test (JMP v.12 software; SAS Inst., Cary, NC, USA). Significant differences between results were estimated at *P* < 0.05.

## 3. Results

### 3.1. Isolation and Host Range Determination of Bacteriophages

From the 127 environmental samples tested, seven phages, specific to* E. coli* O157:H7, were isolated and designated as P-1 to P-7. The isolated phages formed medium-sized (ca. 0.3–0.5 mm in diameter) clear plaques on the lawn of its host bacteria (Table 2).

Host range of the isolated phages was determined against 55* E. coli* O157:H7 isolates (ATCC and WT). All the isolated phages formed clear (+++) spots on the lawn of 49 (89%)* E. coli* O157:H7 isolates, indicating strong lytic activity and a wide host range (Table 1). However, phage P-6 did not lyse isolates RF4 and JF4, and phages P-3 and P-4 did not lyse isolate JF4. All seven phages formed turbid (++) spots against the isolate TE1 and showed no reaction (—) against the WT isolates EF2, RF6, RF7, SW3, and TW3 (Table 1).

### 3.2. Bacteriophage Morphology

**Figure 2:**
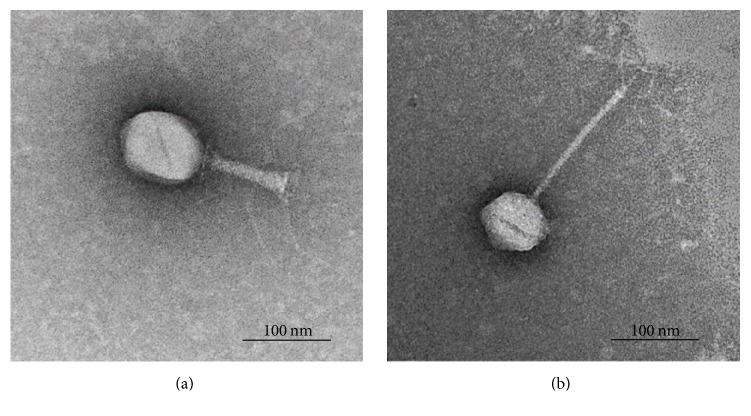
Transmission electron micrograph of phages P-1, P-2, and P-5 belonging to Myoviridae family (a), and P-3, P-4, P-6, and P-7 categorized as Siphoviridae family (b). Scale bar represents 100 nm.

The electron micrographs showed that phages P-1, P-2, and P-5 had an icosahedral head (about 89 nm diameter) and a short contractile tail (ca. 115 nm) with a base plate and several tail fibers (Figure 2(a)). The overall morphology indicated that these phages were T4-like phages, belonging to the Myoviridae family of the Caudovirales order. Phages P-3, P-4, P-6, and P-7 had a hexagonal head (approximately 89 nm diameter) with an extremely long flexible tail (ca. 198 nm) and no visible collar or terminal knobs (Figure 2(b)). They were identified as the Siphoviridae family phages of the Caudovirales order.

### 3.3. Bacteriophage Stability at Acidic and Alkaline pH, at High Temperatures, and in Cold Storage

**Figure 3:**
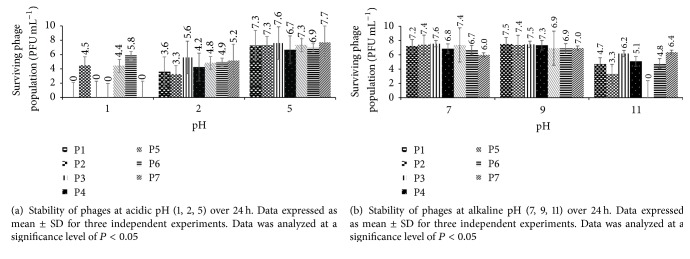


All the isolated phages were tested against a wide pH range (1–11), over a period of 24 h, to determine their stability under acidic and alkaline conditions (Figures 3(a) and 3(b)). In the acidic pH range (1–5), phages exhibited the most stability at pH 5, with minimal loss in viability (0.1–1.7 logs PFU mL^−1^) after 2 h of incubation. Of the seven phages tested, three (P-2, P-5, and P-6) survived at pH 1 over a period of 24 h, with a population of 4.4–5.9 logs PFU mL^−1^ (Figure 3(a)), while P-3 survived at pH 1 for 1 h. All the phages survived at pH 2 over a period of 24 h, with limited activity loss (0.2–3.7 logs PFU mL^−1^) after 1 h of incubation (Figure 3(a)). Among all the phages, P-3 was the most stable at pH 2, with no significant (*P* < 0.05) loss in viability (0.9 logs PFU mL^−1^) after 24 h of incubation. All the phages survived well at pH 7 and 9 with no significant (*P* < 0.05) loss in viability (0.1–1.9 logs PFU mL^−1^) after 24 h of incubation (Figure 3(b)). Phages were also stable at pH 11 for up to 12 h and had minimal reduction (0.8–2.3 logs PFU mL^−1^) in titers after 24 h of incubation (Figure 3(b)). Among all the seven phages, P-7 was the most stable at pH 11 with slight reductions in population (0.4–1.6 PFU mL^−1^) during the 24 h period.

**Figure 4:**
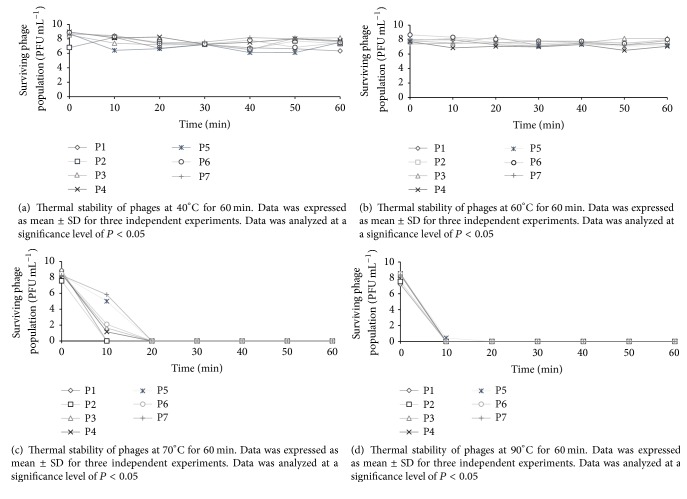


Results from the thermal stability studies revealed that all the tested phages were stable in the 40–60°C temperature range and did not lose viability after 60 min of incubation at the respective temperatures (Figures 4(a) and 4(b)). Phage viability was significantly (*P* < 0.05) affected at higher temperatures of 70 and 90°C (Figures 4(c) and 4(d)). At 70°C, the populations of phages P-5 and P-7 were reduced by 2.5 and 3.3 logs PFU mL^−1^, respectively, after 10 min incubation. Phages P-3, P-4, and P-6 showed a population reduction between 6.4 and 7.3 logs PFU mL^−1^, whereas phages P-1 and P-2 showed no viability after 10 min. All the other phages lost viability after 20 min of incubation at 70°C (Figure 4(c)). At 90°C, no viable phages were detected after 10 min of incubation (Figure 4(d)).

**Figure 5:**
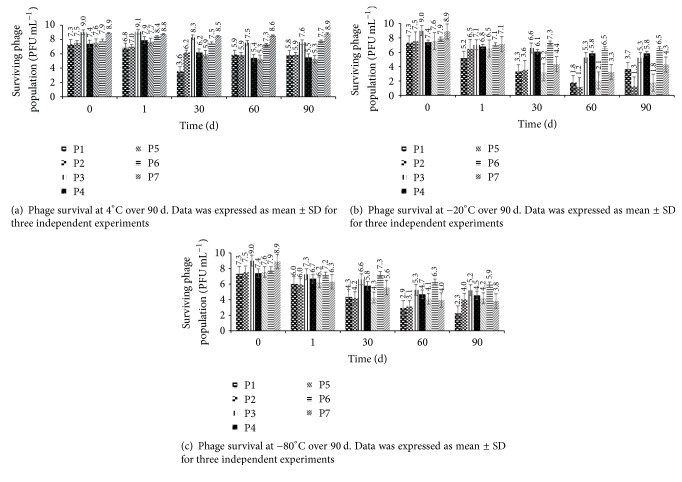


Phages were also tested for cold storage stability and found to be more stable at refrigeration temperature (4°C) than at frozen temperatures (−20 and −80°C) over a period of 90 d (Figures 5(a), 5(b), and 5(c)). At 4°C, minimal reduction in phage populations (0.1–2.3 logs PFU mL^−1^) was observed over a period of 90 d. Phages P-6 and P-7 were the most stable, with minor (0.6 logs PFU mL^−1^) loss in titers during the 90-day storage (Figure 5(a)). At −20°C, all the phages were stable at day 1 with a slight decrease (0.6–2.1 logs PFU mL^−1^) in population (Figure 5(b)). Phages P-3, P-4, and P-6 maintained their titers over the 90-day period. The other phages showed a decrease in population (4.0–6.3 logs PFU mL^−1^) but maintained their viability over a period of 90 d (Figure 5(b)). Phages P-1, P-2, and P-5 showed better survival at −80°C than at −20°C. All the other phages showed a decrease in their population (between 2.0 and 5.1 logs PFU mL^−1^) after 90-day storage (Figure 5(c)). Compared to the other phages, P-6 was the most stable at all cold storage temperatures with a slight decrease (0.6–2.0 logs PFU mL^−1^) in population after 90-day storage.

### 3.4. Phage Adsorption and One-Step Growth Kinetics

**Figure 6:**
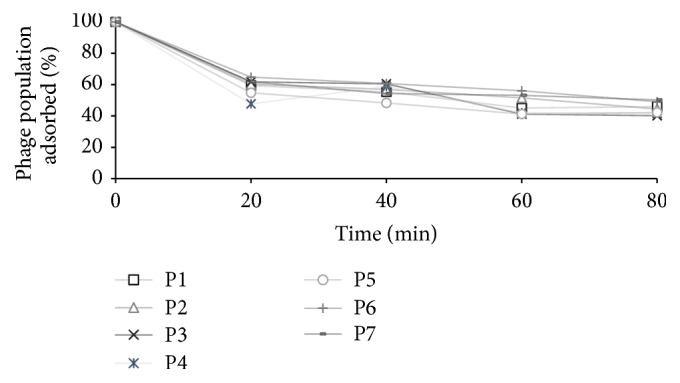
Rate of phage adsorption to host bacterial surface.

Phage growth properties were determined by one-step growth kinetics and phage adsorption rate. Results from the adsorption study revealed that, for all the phages, it took 20 min for approximately 41% of the initial population, and 80 min for approximately 55% of the initial population, to be adsorbed to the host cell surface (Figure 6). Of the seven phages, P-4 had the fastest adsorption rate taking only 20 min for 52% of the population to be adsorbed to the host cell surface. On the other hand, P-3 showed the maximum adsorption rate (60% population adsorbed) after 80 min of incubation.

**Figure 7:**
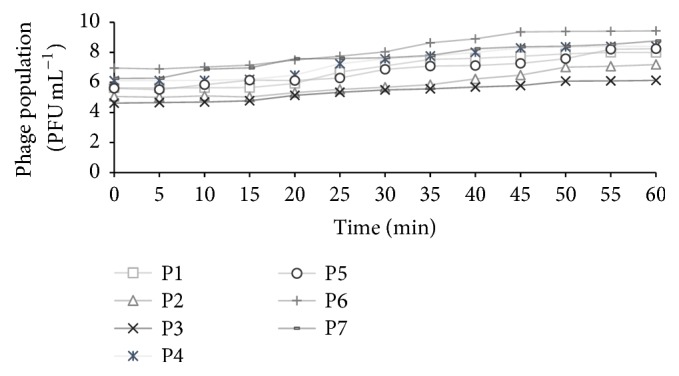
Experimental one-step growth curves of phages over a period of 60 min.

**Figure 8:**
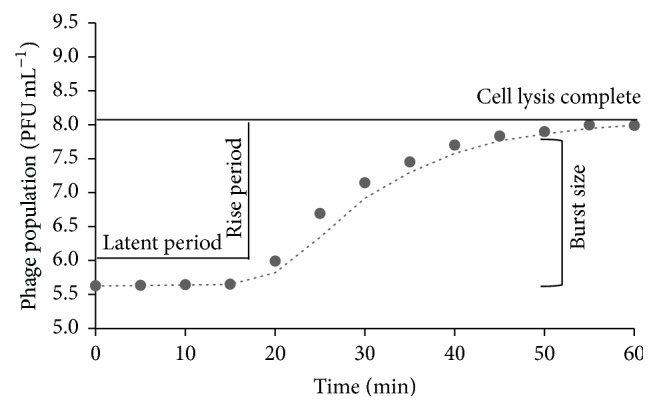
Predicted growth curve of phage P-1 using the 4-parameter sigmoidal function to obtain latent period (21 min), rise period (27 min), and burst size (224 virions per infected cell).

One-step growth kinetics of phages, observed experimentally over a period of 60 min, is shown in Figure 7. Phage growth parameters were calculated using the equation described previously. A representative, predicted one-step growth curve of phage P-1 is shown in Figure 8. Results revealed that phages belonging to the Siphoviridae family (P-3, P-4, P-6, and P-7) had a very short latent period of 12–23 min. On the other hand, the Myoviridae phages (P-1, P-2, and P-5) had a slightly longer latent period of 20–30 min. The rise period was between 19 and 40 min and the burst size was between 89 and 631 virions per infected cell for all the phages.

## 4. Discussion

Several studies have demonstrated the efficacy of bacteriophages against foodborne pathogens; however, limited information is available on their general characteristics and physiology. At the same time, the stability of bacteriophages under various stress conditions, such as acidic and alkaline pH, high temperatures, and cold storage, has not been very well studied. Phage application in the food industry or food-animal industry requires them to be stable in acidic, alkaline, and high or low temperature conditions. For example, food-animal production practices, such as high temperature processing of animal feed, acidic or alkaline environment of the animal GI tract, or antimicrobials used at the farm level, could affect the viability of phages during application. Similar challenges exist at the food-processing level such as high temperature processing, fermentation, and high pressure processing. Additionally, use of multiple-hurdle control strategies is gaining popularity, where phages with high lytic activity may be used in combination with commercial sanitizers, which would require them to be stable and active in concert with the sanitizers. It is therefore important to determine the stability of bacteriophages in adverse conditions to select highly effective phages for application in the food industry and food-animal industry. In the present study, physiomorphological characteristics of bacteriophages, isolated from beef cattle environment, and their stability at a wide pH and temperature range along with their growth and lytic properties were determined to identify and select effective phages against* E. coli* O157:H7.

All the isolated phages showed high lytic activity against 50 isolates of* E. coli* O157:H7, including the WT isolates (Table 1), suggesting that they have a broad host range. Studies have shown that phages specific to one* E. coli* O157:H7 strain can also infect other O157:H7 strains [39, 57, 58]. In a study by Raya et al. [39], phage AR1 lysed all O157:H7 tested strains, while in another study [59], phage CEV1 infected 17 of the tested 19 strains of* E. coli* O157:H7. Results from the current study are similar to these studies, revealing that isolated phages are virulent against a wide range of* E. coli* O157:H7 isolates with high target specificity.

Phages in the current study were also stable at a wide pH range (1–11) compared to the phages in other studies [49, 60]. Niu et al. [49] revealed that at pH 3 the titer of* E. coli* O157:H7 phage, AKFV33, dropped by 1.9 logs PFU mL^−1^ after 15 min and was undetectable after 2 h. In the current study, 3 of the 7 phages survived at pH 1, while all phages survived at pH 2 and 5 after 24 h, with minimal loss in viability. It is possible for phages to acquire nonreversible mutations at low pH [61–63], which could explain the survival of phages at low pH in the present study. Strack et al. [61] showed a linear relationship between phage mutation rate and incubation at low pH, suggesting that phages can acquire mutation to survive acidic environment. Phages in the current study were also stable at alkaline pH (7–11), showing no loss at pH 7 and 9 and minimal loss in titer at pH 11. In earlier studies by the authors, isolated phages were found to be stable in water (pH 7) for a period of 30 d (unpublished data). Reductions in phage titers at pH 11 could be due to the dissociation of the capsid protein, caused by high concentrations of hydrogen and hydroxyl ion in the solution [64]. The ability of isolated phages to survive extreme pH conditions could be utilized in a number of applications in the food and animal industry. Survival at low pH could be used to control foodborne pathogen colonization in the animal GI tract (pH 1–5), through oral administration [57, 65, 66]. It can also be used as biocontrol in acidic foods such as fruit juices, fermented products, and pickles. Furthermore, their ability to survive alkaline pH could be utilized during the multiple-hurdle approach in processing facilities or on food-contact surfaces [67, 68].

Studies have shown that high temperature inactivates phages due to nucleic acid and protein denaturation [50, 69]. Yamaki et al. [50] observed that Myoviridae phages drastically lost phage activity (3.5 logs PFU mL^−1^) after 60 min of incubation at 60°C. However, in the current study, no significant decrease in phage activity was observed at 60°C for 60 min. Additionally, five of the seven phages (P-3, P-4, P-5, P-6, and P-7) also survived at 70°C for 10 min. Different phage strains behave differently to heat treatments [70], which could explain the difference in results in the two studies. Furthermore, Yamaki et al. [50] used the phage infecting* Morganella morganii*, which is sensitive to high temperature [71] and may therefore be sensitive to high temperatures. Additionally, some phages can develop heat resistance due to mutations or strong protein interactions [72], explaining the survival of phages at high temperatures in the current study.

Bacteriophage viability at refrigerated and frozen temperatures was determined to ensure their survival during storage or shipment. All the phage isolates maintained their viability over a storage period of 90 d at 4, −20, and −80°C. Previous studies have revealed that tailed phages are resistant to refrigeration temperatures and can retain viability for more than 10–12 years at 4°C [73, 74]. In the current study, phages survived very well at 4°C, with minimal activity loss during the 90-day storage. However, compared to 4°C, phages were less stable at −20 and −80°C and showed a decrease in population after 90-day storage. Compared to −20°C, phages P-2 and P-5 showed better survival at −80°C, while other phages showed similar survival trend at both storage temperatures. Loss in phage titers at −20 and −80°C during 90-day storage could be due to the formation of ice crystals at frozen temperatures [75, 76]. For long-term storage, it is recommended that phages be maintained at −80°C [74], which could be true for phages P-2 and P-5 in the current study.

The efficacy of phages to infect bacterial cells was measured using phage growth kinetics and adsorption rate. Isolated phages had a long rise period (19–40 min) and a short latent period (12–30 min) with a large burst size (89–631 virions per infecting cell), suggesting that they had a high progeny production rate. The average burst size of tested phages was about 329 phages per infected cell, which is more than the typical range of 50–100 PFU cell^−1^ for many Myoviridae and Siphoviridae phages [77, 78]. Furthermore, the Siphoviridae phage, P-7, had the largest burst size of 631 virions per infected cell, which is much larger than other Siphoviridae phages [77, 79]. The latent period and rise period of the characterized phages were similar to those of the other Myoviridae and Siphoviridae phages [40, 78–81]. Bacteriophages with a short latent period and large burst size may have a selective advantage over other phages due to high lytic activity [78]. These characteristics of isolated phages could therefore be applied to develop control strategies for the transmission and survival of* E. coli* O157:H7 in the food chain. However, one concern with this approach is the emergence of phage-resistant bacteria, particularly with application of phages in animals or on the farm. Use of phage cocktails that are regularly updated with new or different phages could potentially address the issue, by maintaining selective pressure on bacterial host [43, 82].

## 5. Conclusions

This study provides a better understanding of the infection kinetics of seven phages, targeting* E. coli* O157, and their survivability under various stress conditions. This information will afford the ability to determine the application of phage-based interventions. Bacteriophages isolated in this study showed a wide range of host specificity towards* E. coli* O157:H7 isolates, along with high lytic activity, and pH and thermal stability and could therefore be used as biocontrol agents in the food industry.

## Figures and Tables

**Table 1:** Host range^*∗*^ of phages against *E. coli* O157:H7 isolates.

Strains	P-1	P-2	P-3	P-4	P-5	P-6	P-7
ATCC 43895	+++	+++	+++	+++	+++	+++	+++
ATCC 43888	+++	+++	+++	+++	+++	+++	+++
O157:H7 RF1	+++	+++	+++	+++	+++	+++	+++
O157:H7 RF2	+++	+++	+++	+++	+++	+++	+++
O157:H7 RF3	+++	+++	+++	+++	+++	+++	+++
O157:H7 RF4	+++	+++	+++	+++	+++	—	+++
O157:H7 RF5	+++	+++	+++	+++	+++	+++	+++
O157:H7 RF6	—	—	—	—	—	—	—
O157:H7 RF7	—	—	—	—	—	—	—
O157:H7 RF8	+++	+++	+++	+++	+++	+++	+++
O157:H7 RF9	+++	+++	+++	+++	+++	+++	+++
O157:H7 RF10	+++	+++	+++	+++	+++	+++	+++
O157:H7 RF11	+++	+++	+++	+++	+++	+++	+++
O157:H7 RF12	+++	+++	+++	+++	+++	+++	+++
O157:H7 RF13	+++	+++	+++	+++	+++	+++	+++
O157:H7 RF14	+++	+++	+++	+++	+++	+++	+++
O157:H7 RF15	+++	+++	+++	+++	+++	+++	+++
O157:H7 SF1	+++	+++	+++	+++	+++	+++	+++
O157:H7 SF2	+++	+++	+++	+++	+++	+++	+++
O157:H7 SF4	+++	+++	+++	+++	+++	+++	+++
O157:H7 SF5	+++	+++	+++	+++	+++	+++	+++
O157:H7 SF7	+++	+++	+++	+++	+++	+++	+++
O157:H7 SF8	+++	+++	+++	+++	+++	+++	+++
O157:H7 SF10	+++	+++	+++	+++	+++	+++	+++
O157:H7 SF12	+++	+++	+++	+++	+++	+++	+++
O157:H7 SF14	+++	+++	+++	+++	+++	+++	+++
O157:H7 SF15	+++	+++	+++	+++	+++	+++	+++
O157:H7 SW3	—	—	—	—	—	—	—
O157:H7 TW3	—	—	—	—	—	—	—
O157:H7 TE1	++	++	++	++	++	++	++
O157:H7 TE2	+++	+++	+++	+++	+++	+++	+++
O157:H7 TF1	+++	+++	+++	+++	+++	+++	+++
O157:H7 TF2	+++	+++	+++	+++	+++	+++	+++
O157:H7 TF3	+++	+++	+++	+++	+++	+++	+++
O157:H7 TF4	+++	+++	+++	+++	+++	+++	+++
O157:H7 TF6	+++	+++	+++	+++	+++	+++	+++
O157:H7 TF7	+++	+++	+++	+++	+++	+++	+++
O157:H7 TF8	+++	+++	+++	+++	+++	+++	+++
O157:H7 TF9	+++	+++	+++	+++	+++	+++	+++
O157:H7 TF10	+++	+++	+++	+++	+++	+++	+++
O157:H7 TF11	+++	+++	+++	+++	+++	+++	+++
O157:H7 TF12	+++	+++	+++	+++	+++	+++	+++
O157:H7 TF13	+++	+++	+++	+++	+++	+++	+++
O157:H7 TF14	+++	+++	+++	+++	+++	+++	+++
O157:H7 RF15	+++	+++	+++	+++	+++	+++	+++
O157:H7 XW5	+++	+++	+++	+++	+++	+++	+++
O157:H7 JF4	+++	+++	—	—	+++	—	+++
O157:H7 LF4	+++	+++	+++	+++	+++	+++	+++
O157:H7 KF10	+++	+++	+++	+++	+++	+++	+++
O157:H7 JEQ1	+++	+++	+++	+++	+++	+++	+++
O157:H7 JF6	+++	+++	+++	+++	+++	+++	+++
O157:H7 KF7	+++	+++	+++	+++	+++	+++	+++
O157:H7 LF5	+++	+++	+++	+++	+++	+++	+++
O157:H7 EF2	—	—	—	—	—	—	—

^*∗*^Based on the degree of clarity on the bacterial lawn, the spots were differentiated into three categories: clear (+++), turbid (++), or no reaction (—).

**Table 2:** Titer (PFU mL^−1^) and plaque size (mm) of isolated phages.

Phages	Titer (PFU mL^−1^)	Plaque size (mm)
P-1	7.9	0.3
P-2	7.4	0.4
P-3	7.8	0.5
P-4	7.7	0.5
P-5	7.5	0.4
P-6	8.0	0.5
P-7	7.5	0.5
